# LncRNA HOTAIR down-expression inhibits the invasion and tumorigenicity of epithelial ovarian cancer cells by suppressing TGF-β1 and ZEB1

**DOI:** 10.1007/s12672-023-00846-5

**Published:** 2023-12-09

**Authors:** Yufu Zhou, Yunjie Zhang, Yidan Shao, Xiaoli Yue, Yifan Chu, Cuiping Yang, Dengyu Chen

**Affiliations:** 1 Department of Radiotherapy, The First Affiliated Hospital of Bengbu Medical College, Bengbu, 233000 Anhui People’s Republic of China; 2 Department of Microbiology, Bengbu Medical College, Bengbu, 233030 People’s Republic of China; 3 Anhui Key Laboratory of Infection and Immunity, Bengbu Medical College, Bengbu, 233030 People’s Republic of China; 4 Department of Ophthalmology, The First Affiliated Hospital of Bengbu Medical College, Bengbu, 233000 Anhui People’s Republic of China; 5 Laboratory Center for Morphology, Bengbu Medical College, Bengbu, 233030 People’s Republic of China; 6 Department of Gastroenterology, Ruijin Hospital, Shanghai Jiaotong University School of Medicine, Shanghai, 201801 People’s Republic of China

**Keywords:** Epithelial ovarian cancer, lncRNA HOTAIR, Tumorigenicity, TGF-β1, ZEB1

## Abstract

**Background:**

Epithelial ovarian cancer (EOC) is a pathological type with a higher mortality rate among gynecological cancers today. Long-chain noncoding RNAs (lncRNAs) can regulate the transcription and expression of cellular genes. However, the downstream molecules regulated by lncRNA HOTAIR have not been well studied. The effects of downregulated lncRNA HOTAIR on EOC invasiveness and tumorigenicity in nude mice, along with TGF- β1 and ZEB1 in epithelial ovarian cancer cells, need to be investigated in further research.

**Results:**

RT-qPCR was used to detect lncRNA HOTAIR and TGF-β1 and ZEB1 mRNA expression in EOC SKOV3 cells. The expression of lncRNA HOTAIR in SKOV3 cells transfected with the recombinant shHOTAIR interference plasmid was significantly lower than that of the negative control. Compared with the negative control, the matrix gel invasion ability of shHOTAIR SKOV3 cells in vitro and their tumorigenicity in nude mice were significantly reduced. Moreover, compared with the control, the expression of ZEB1 protein in shHOTAIR-SKOV3 xenograft tumors was significantly reduced. Downregulation of lncRNA HOTAIR expression significantly reduced TGF-β1 and ZEB1 mRNA expression, but increased the expression of E-cadherin mRNA. In summary, downregulated lncRNA HOTAIR in EOC SKOV3 cells transfected with shHOTAIR can inhibit TGF-β1, reduce ZEB1, increase E-cadherin, and significantly reduce the invasiveness and tumorigenicity of ovarian epithelial cancer SKOV3 cells.

**Conclusions:**

These results suggest that the lncRNA HOTAIR may be an effective target for the treatment of human EOC.

**Supplementary Information:**

The online version contains supplementary material available at 10.1007/s12672-023-00846-5.

## Introduction

Epithelial ovarian cancer (EOC) originates from the ovary epithelium. It is a common pathological type of ovarian cancer, and its incidence rate accounts for 70% of ovarian malignant tumors [1]. Epithelial ovarian cancer patients are mostly middle-aged and elderly women. EOC also has different pathological types, most of which are serous adenocarcinomas. The specific etiology of epithelial ovarian cancer is still unclear, and may be related to genetic factors, endometriosis, and other factors. Due to the hidden anatomical location of the primary lesion in epithelial ovarian cancer, most ovarian cancers are only discovered when symptoms appear in the middle to late stages of tumor development. At this point, most epithelial ovarian cancer has directly spread to bring about abdominal metastasis, which can result in lower five-year survival rates of epithelial ovarian cancer patients [2].

Nowadays, long-chain noncoding RNAs (lncRNAs) have been found to regulate cell gene expression and play a significant role in tumor growth, invasion, and metastasis. The target of its action needs in-depth research to promote cancer prevention and control strategies. HOTAIR is a lncRNA HOX antisense intergenic RNA. It participates in cell proliferation and migration. It was also involved in epithelial-mesenchymal transformation (EMT) in normal tissue and tumors, and lncRNA HOTAIR has potential for cancer targeted therapy [3, 4]. To predict and validate the target sites of lncRNA HOTAIR, first, to identify relevant targets and contact networks, we constructed an shRNA interference vector with low expression of lncRNA HOTAIR and transfected it into EOC SKOV3 cells. After that, we detected its impact on lncRNA HOTAIR expression. Second, we studied the inhibitory effects of low expression of lncRNA HOTAIR after shRNA downregulation on the invasion of matrix gel in epithelial ovarian cancer cells and nude mouse tumorigenicity, and further investigated the related molecular mechanisms.

## Materials and methods

### Cell line and animal

SKOV3, a human epithelial ovarian cancer cell line, was purchased from the Shanghai Cell Institute of the Chinese Academy of Sciences. BALB/c nude mice, which were female, three to four weeks old and fifteen to twenty grams in weight, were selected and bought from Yangzhou University, China.

### Reagents

These reagents were purchased from the next companies. The quantitative PCR kits, Roche Company. Molecular cloning related reagents, Promega Company, USA. RNA extraction kit and liposome 2000, Invitrogen Company. G418, Sigma Company. Transwell Chambers, Corning Company, USA. Matri Gel, BD Company. RT, PCR kits, human zinc finger E-Box binding homeobox 1 antibody, Shanghai Bioengineering Co., Ltd. Immunohistochemistry detection kits, Shanghai Bioengineering Co., Ltd.

### Reverse transcription PCR and RT-qPCR

The checked RNA was drawn from SKOV3 or transfected SKOV3 cells and transplanted tumor tissues in tumor-bearing mice. Specific and random primers for reverse transcription were used to amplify the lncRNA HOTAIR and the mRNAs of TGF-β1, ZEB1 and E-cadherin for RT-PCR and quantitative PCR. Meanwhile, human β-actin was used as an internal reference for testing. The lncRNA HOTAIR(Gene ID: 100124700)primers, were as followed: up 5ʹ—GGTAGAAAAAGCAA CCACGAAGC—3ʹ, down5ʹ—TTGGGGAAGCATTTTCTGAC—3ʹ, product 744 bp. Long-chain noncoding RNA HOTAIR was checked by reverse transcription PCR and quantitative PCR. The primers for human E-cadherin, were up 5ʹ—TACACTGCCCAGGAGCCAGA—3ʹ, down 5ʹ—TGGCACCAGTGTCCGGATTA—3ʹ, and the obtained product 103 bp. The primers of human ZEB1, up 5ʹ—TAAAGTGGCGGTAGATGGTA—3ʹ, down 5ʹ—CTGTTTGTAGCGACTGGATT—3ʹ, product 258 bp. The human TGF-β1 primers, were up 5ʹ—AGTGGACATCAACAACGGGTTCAC—3ʹ, down 5ʹ—ATGAGAAGCAGGAAAGGCCG—3ʹ. The primers for human β-actin, were up 5ʹ—GGACTTCGAGCAAGAGATGG—3ʹ, down 5ʹ—AGCACTGTGTTGGCGTACAG—3ʹ, and the obtained product 234 bp. According to the kit instructions, TGF-β1, ZEB1, and E-cadherin mRNA were detected by quantitative PCR. △Ct and the relative expression level were calculated and analyzed.

### shRNA vector construction and transfection

First, the pSUPER-EGFP1 (enhanced green fluorescent protein 1) vector was chosen and used as the shRNA vector. Second, based on the methods of reference literature [5], the construction of the shRNA-lncRNA HOTAIR pSUPER-EGFP1 vector was conducted and proved to be correct by means of glittering gene sequencing.

The lncRNA HOTAIR shRNA sequences were as follows: Forward 5ʹ—GATCCCCGAACGGGAGTACAGAGAGATTCAAGA GATCTCTCTGTACTC CCGTTCTTTTTGGAAA—3ʹ.

The constructed pSUPER-EGFP1-shRNA-lncRNA HOTAIR (shHOTAIR) and control pSUPER-EGFP1-scramble were separately transfected into EOC SKOV3 cells. After that, the stably transfected cell lines were chosen following the plasmid biological characteristics, which included green fluorescent protein (GFP) expression and G418 resistance, so as to conduct the next functional experiments.

### Transwell invasion test

Based on the reference literature methods [5], the transwell Matrigel invasion assay was carried out in order to check cell invasion capability.

### Tumorigenicity detection

After 7 days, the purchased nude mice were raised, and shHOTAIR SKOV3 and scramble-SKOV3 1 × 10^6^ cells were inoculated into their back subcutaneous tissue. In each group, six nude mice were tested, with daily monitoring of tumor growth. Sixty days later, the tumors were removed from the nude mice.

### HE and IHC detection

The stripped xenograft tumor portions were embedded in paraffin. Afterward, hematoxylin–eosin staining (HE staining) was performed for histopathological observation. ZEB1 protein in xenograft tumors was checked by immunohistochemistry (IHC) detection. Based on the documentary methods [6], IHC detection results were determined. The nucleus stained with ZEB1 protein shows brown granules indicating positivity.

### RT-qPCR in xenograft tumors

On the basis of the aforementioned RT-qPCR, TGF-β1, ZEB1 and E-cadherin mRNA of nude mouse tumors were checked.

### Statistical analysis

These experiments were repeated 3 times. Paired* t* tests were used to analyze the differences between the two groups. A *P* value of < 0.05 was considered statistically significant.

## Results

### LncRNA HOTAIR detection

**Fig.1:**
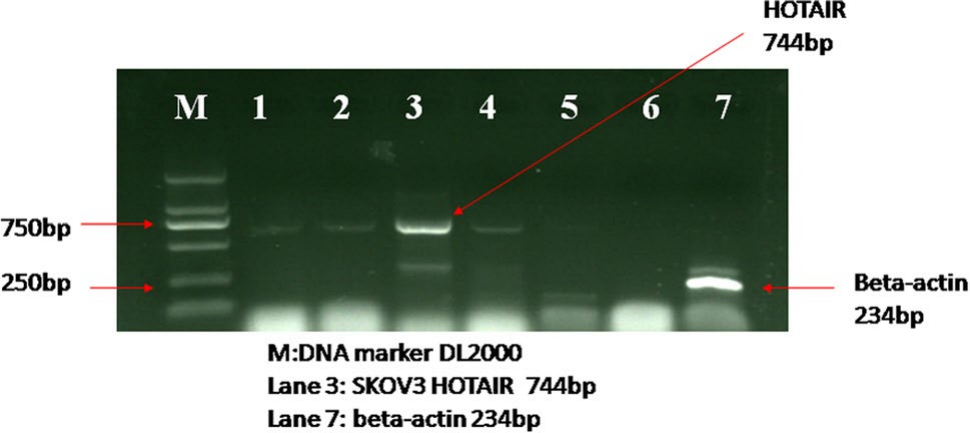
RT-PCR detection results. The DNA maker is located in Lane M. β-Actin is located in Lane 7. The product of lncRNA HOTAIR located in Lane 3

RT-PCR was used to detect the expression of lncRNA HOTAIR in SKOV3 ovary adenocarcinoma cells, Fig. 1. The HOTAIR product in Fig. 1 shows 744 bp, indicating that lncRNA HOTAIR is expressed in epithelial ovarian cancer SKOV3 cells.

### Screening of stably transfected cell clones

**Fig. 2:**
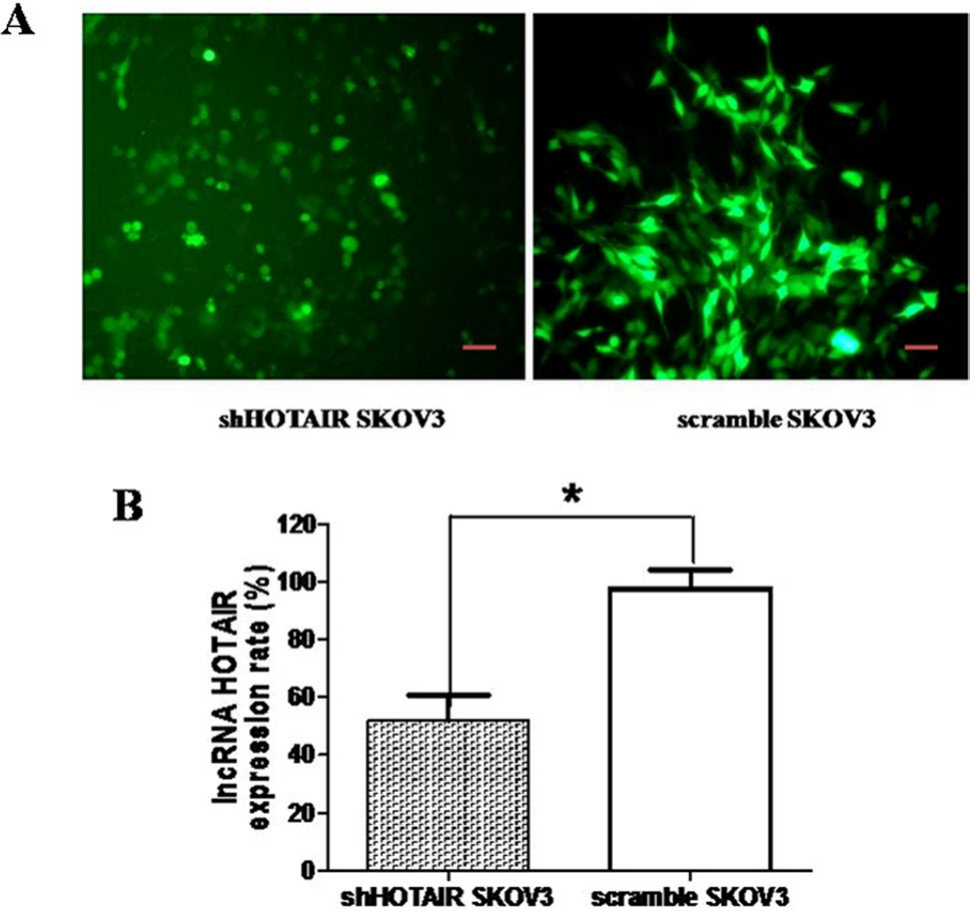
Stably transfected SKOV3 cell clones **A**. By fluorescence microscopy, green fluorescent protein was examined in transfected EOC SKOV3 cells, magnification ×400, scale bar: 50 μm. The cell growth was vigorous in the control group. In contrast, shHOTAIR SKOV3 proliferation was not vigorous and lacked a cluster. B. RT-qPCR result analysis

After transfecting the recombinant plasmids, the cell clones of stably transfected plasmids were chosen and determined. Regarding the expression of the distinctive green fluorescent protein, the stably transfected shHOTAIR SKOV3 cells were screened, as shown in Fig. 2a.

RT-qPCR was used to detect the relative expression of lncRNA-HOTAIR between control scramble and shHOTAIR transfected EOC cells. The relative expression rate of lncRNA HOTAIR in EOC shHOTAIR SKOV3 cells (49 ± 7%) was markedly lower than that the scramble control cells (100 ± 7%) (*P < 0.05), as shown in Fig. 2b. The results indicate that shHOTAIR transfection is effective, and the obtained the downregulated lncRNA HOTAIR EOC SKOV3 cells can be used for functional experiments.

### Invasiveness detection

**Fig. 3:**
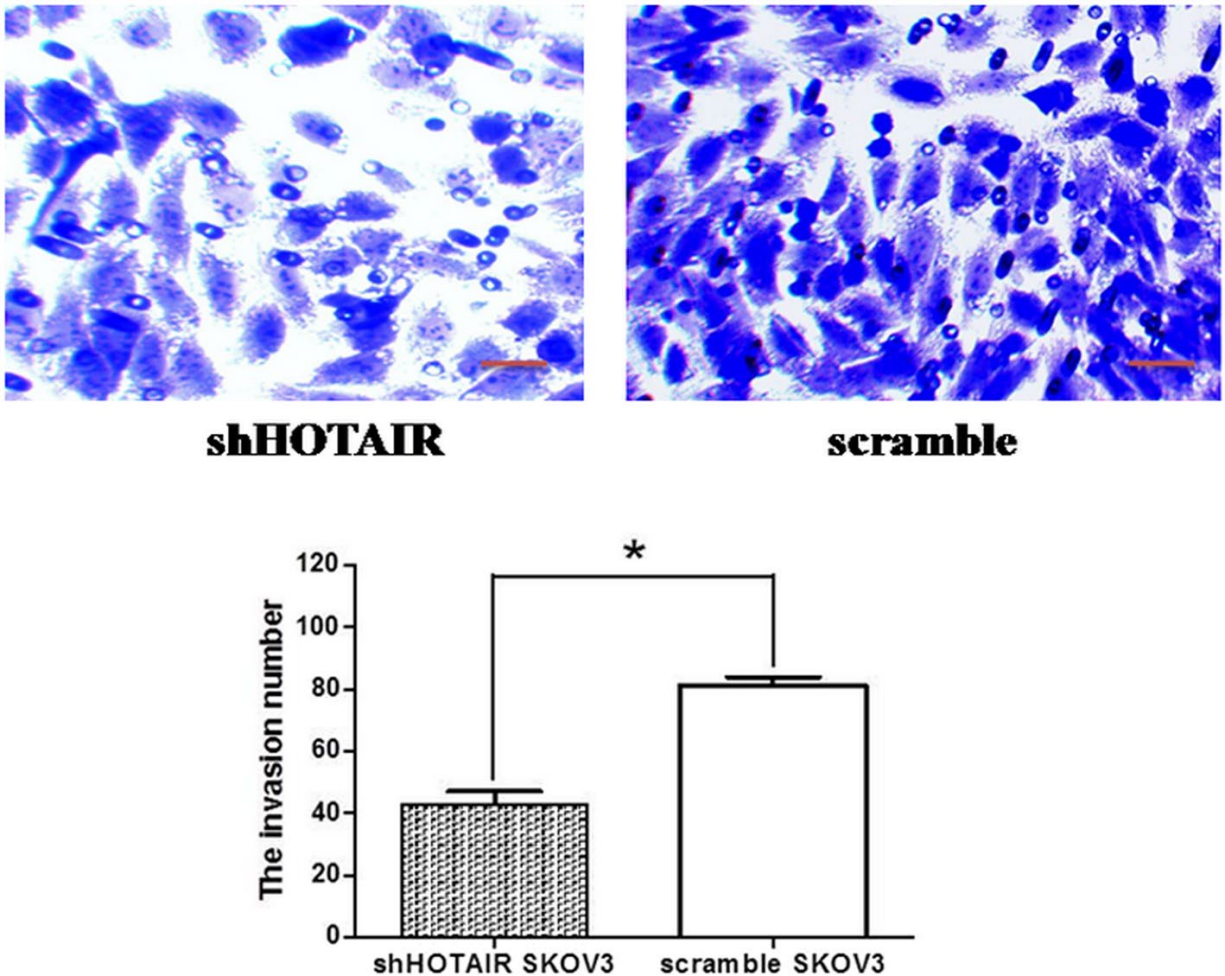
Invasiveness detection The invasion number of shHOTAIR-transfected SKOV3 cells, 43 ± 8 in each observational field, was substantially lower than that of the control. Statistical analysis showed *P* < 0.05. Scale bar: 50 μm

Invasiveness detection of transfected cells is shown in Fig. 3. In each observed field, the invasiveness of shHOTAIR-transfected SKOV3 cells (average 43 ± 8 cells) was substantially lower than that(81 ± 6) of the control scramble SKOV3 cells. The difference was statistically significant (*P* < 0.05), as shown in Fig. 3.

### Experiment with nude mouse

**Fig. 4:**
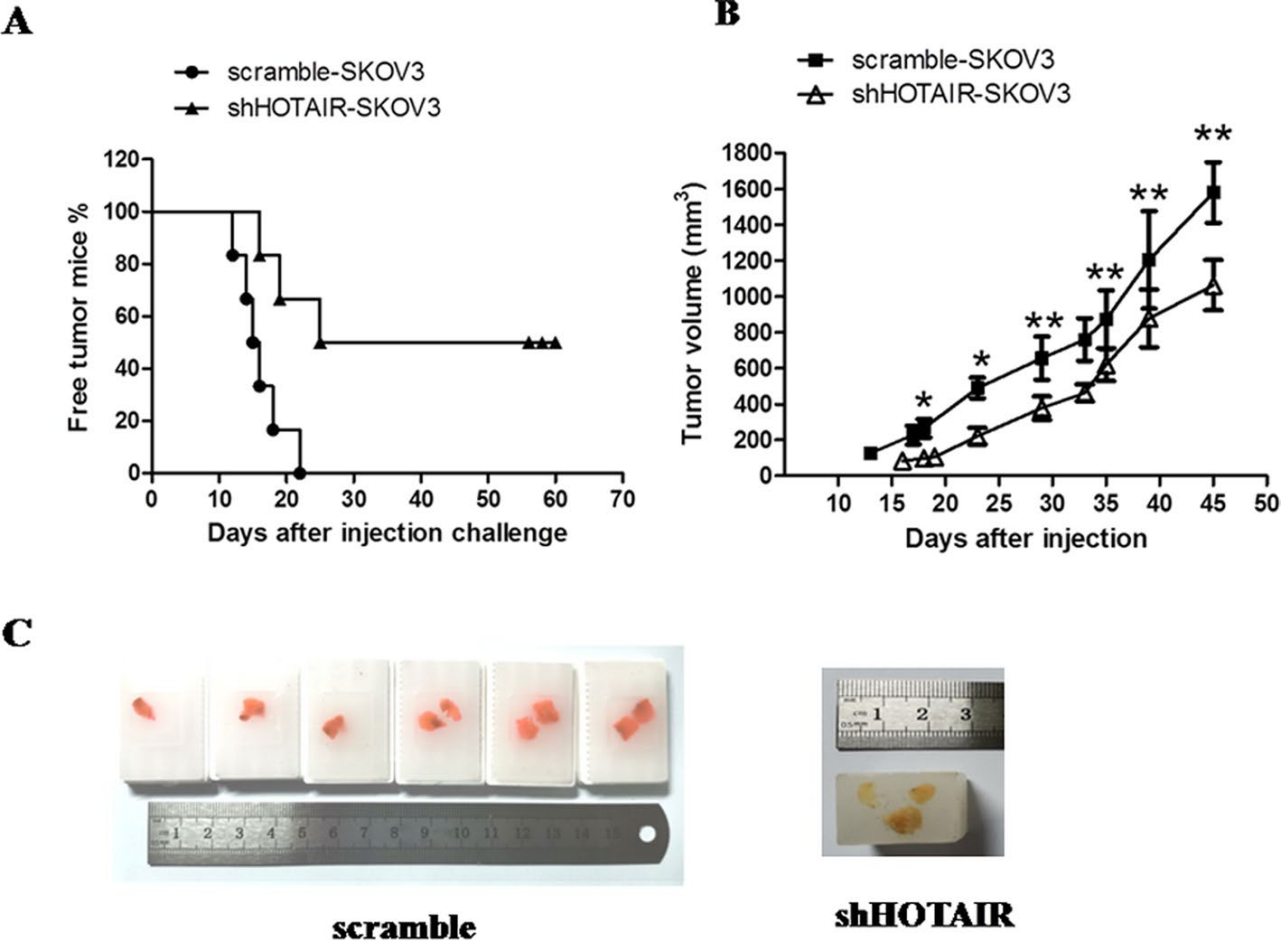
Tumor growth change **A**. Percentage of free tumor mice in various groups. Compared with the negative control group, the occurrence time of tumors in the shHOTAIR-SKOV3 group was significantly delayed. **B**. Tumor volume change. (*P < 0.05 or **P < 0.01). The shHOTAIR-SKOV3 tumors grew slowly and had smaller volumes than those in the control group. **C**. Tumor pictures. The paraffin embedded image of the transplanted tumor mass is presented

Experiments with nude mice strictly complied with relevant ethical standards for animal experiments. The procedures conducted on animals were approved by the Ethics Committee of Bengbu Medical College (Project Number: animal ethics 2022–222). The maximal tumor size/burden permitted by the Ethics Committee is 20 mm in diameter or 2000 mm^3^ in volume. No mouse has violated this standard. The maximal tumor size/burden was not exceeded. The subsequent experiment with nude mice compared the different tumorigenicity of SKOV3 cells transfected with shHOTAIR and control scramble. Differentially transfected SKOV3 cells were subcutaneously injected into the backs of nude mice, and the appearance time of the tumors was observed. In the control group, six nude mice were subcutaneously injected with scramble-SKOV3 cells. Their tumors were formed and observed from 13 to 23 days after injection, and the tumor sizes were slowly and continuously increasing. In the shHOTAIR-SKOV3 group, only three mice developed tumors on days 16, 19, and 25. Compared with the negative control group, the occurrence time of tumors was significantly delayed. The other three nude mice did not develop tumors within 60 days from the beginning to the end of the experiment. The shHOTAIR-SKOV3 tumors grew slowly and had smaller volumes than those in the control group. Between the two groups, the difference in tumor sizes was statistically significant, as shown in Fig. 4. This result indicated a decreasing trend in the tumorigenic capability of ovarian cancer cells in the shHOTAIR SKOV3 group.

### Tumor section staining detection

**Fig. 5:**
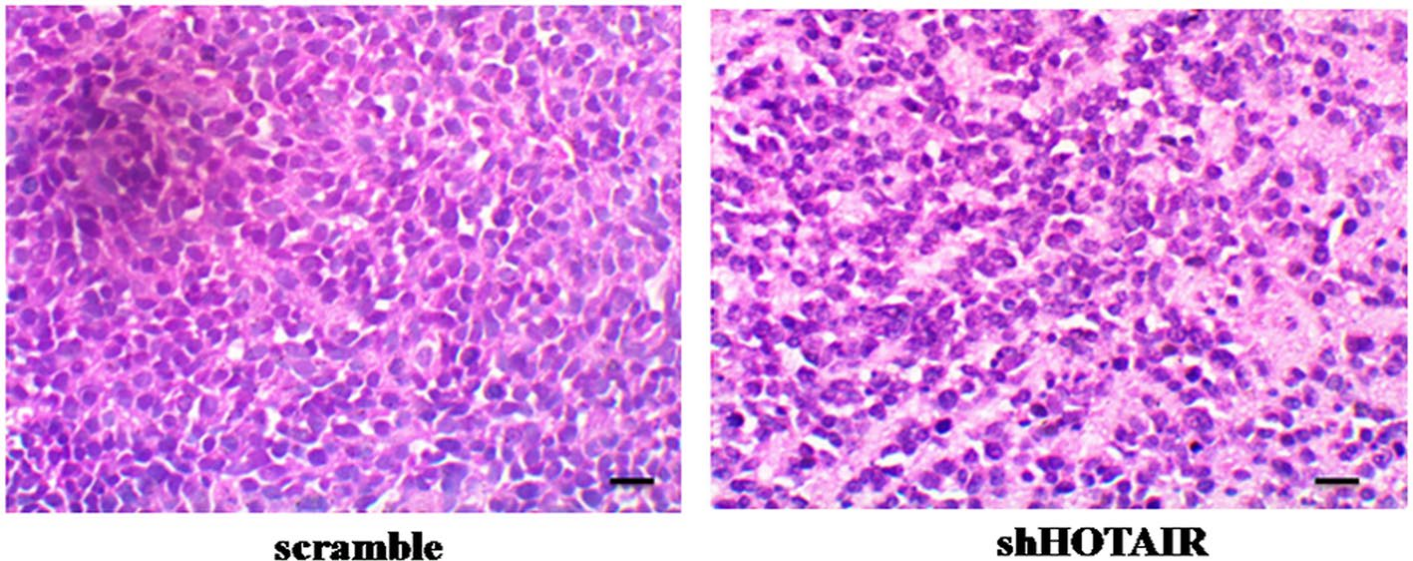
Tumor HE staining Tumor HE staining, magnification×400, scale bar: 50 μm. The tumor cell proliferation in the control scramble SKOV3 group was very active. After HE staining, the tumor cells showed purple-blue nuclei and red cytoplasm under the microscope, with an increase in the ratio of nucleoli to cytoplasm. The nucleus is elliptical or irregularly shaped. However, shHOTAIR SKOV3 cells in tumor tissue have lower proliferative activity

The tumor sections were stained with HE staining and observed under a microscope, and the expression of zeb1 protein in tumor cells was detected by immunohistochemistry. The results of HE staining, shown in Fig. 5, showed that the tumor cell proliferation in the control scramble SKOV3 group was very active. The HE staining results are shown in Fig. 5, showing that the tumor SKOV3 cells in the control group proliferated very actively. After HE staining, the tumor cells showed purple blue nuclei and red cytoplasm under the microscope, with an increase in the ratio of nucleoli to cytoplasm. Tumor cells are generally large in size, with large nuclei, abnormal nuclei, deep staining, and frequent mitotic phases. The nuclei of tumor cells are oval or irregularly shaped. However, the proliferation activity of shHOTAIR SKOV3 cells in tumor tissue is relatively low.

The immunohistochemical detection results showed that the protein expression level of zeb1 in tumor cells of xenografts of shHOTAIR SKOV3 cells inoculated with tumor bearing mice was significantly lower (IHC staining showed a comprehensive score of 2 ± 1) than the protein expression level of zeb1 in the control group (IHC score of 4 ± 1).

**Fig. 6:**
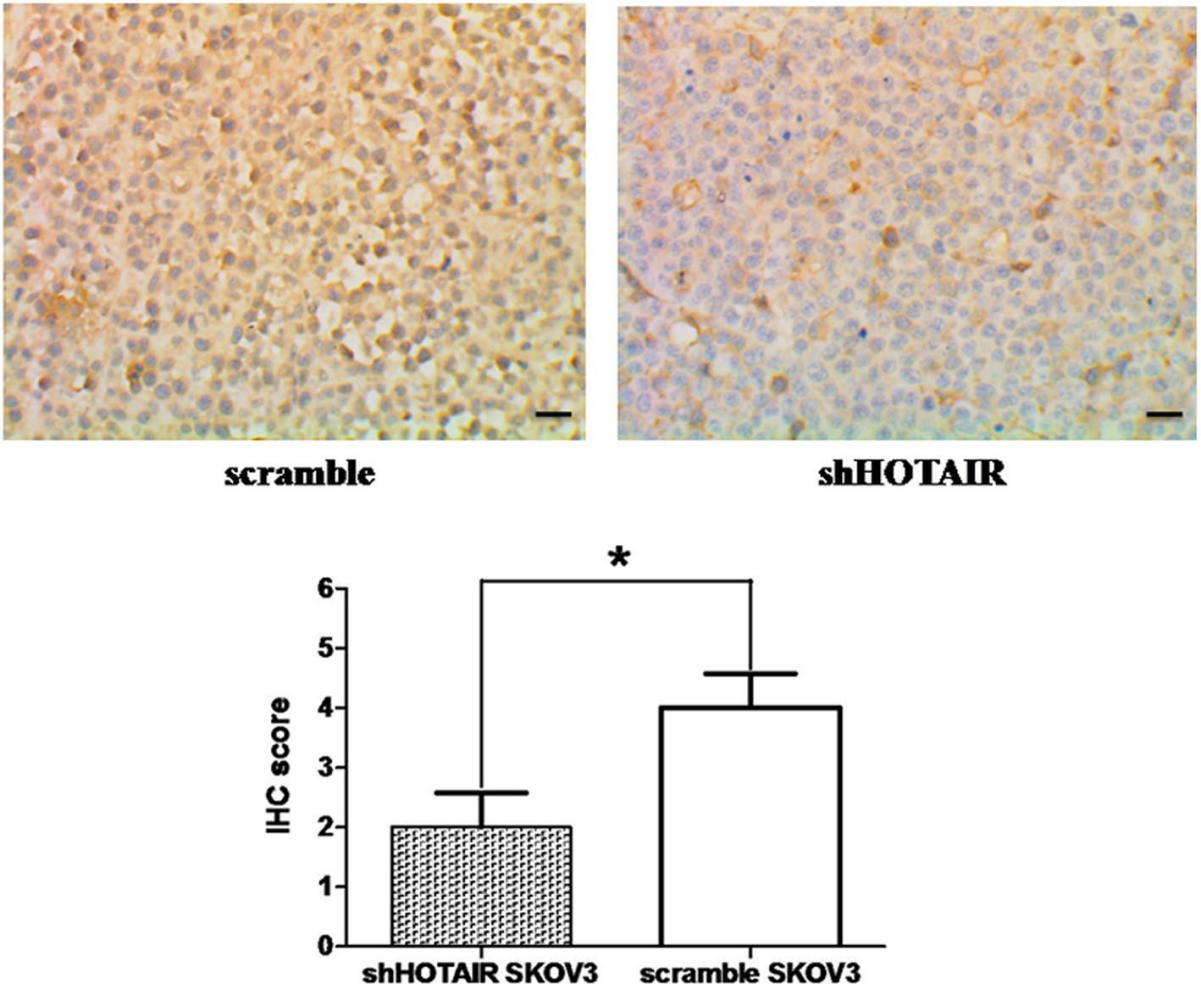
IHC detection. Zeb1 of xenograft tumors was detected by immunohistochemistry, method of SP, magnification×400, scale bar: 50 μm, with statistical significance (*P < 0.05)

The difference in the expression level of ZEB1 protein between the two groups was statistically significant (*P* < 0.05), and the immunohistochemical and statistical figures are shown in Fig. 6. Downregulated lncRNA HOTAIR leads to a decrease in the expression of zeb1 protein in EOC SKOV3 cells.

### RT-qPCR detection

**Fig. 7:**
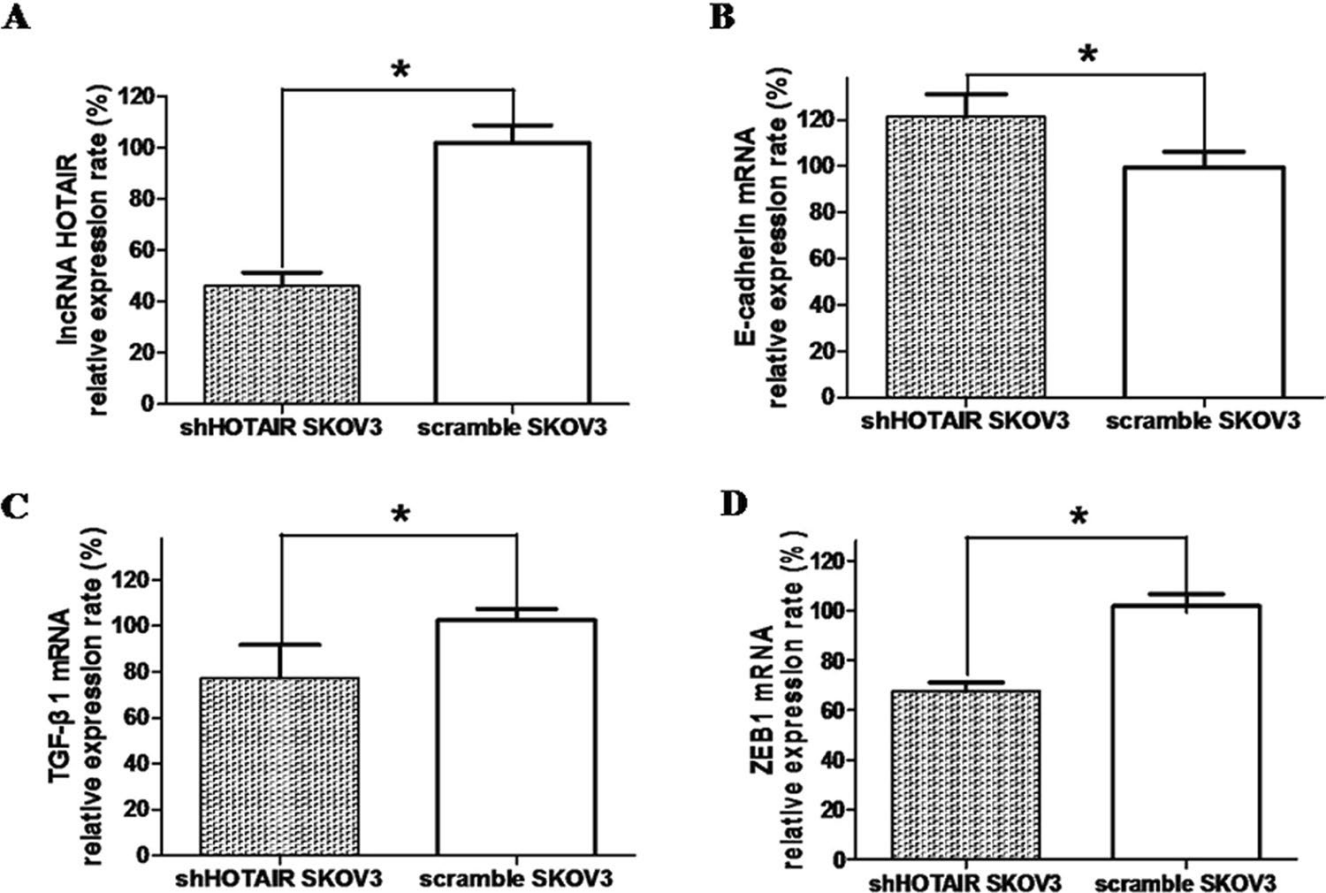
Real-time PCR detection. **A. **LncRNA HOTAIR detection through real-time real-time polymerase chain reaction. **B**. RT-qPCR detection of E-cadherin mRNA. **C**. Detection of transforming growth factor- β1mRNA by RT-qPCR. **D**. Real-time PCR detection of Zeb1 mRNA. Real-time results showed that the expression of E-cadherin mRNA in shHOTAIR SKOV3 cells from injected tumor-bearing mice was higher than that in control, but lncRNA HOTAIR, TGF-β1, and Zeb1 were lower than that of the control group. The difference was statistically significant (*P < 0.05)

As shown in Fig. 7a, the relative expression rate of the lncRNA HOTAIR in shHOTAIR xenograft tumors (45 ± 11%) was obviously lower than the control group. Detection of E-cadherin, TGF-β1 and zeb1 mRNA expression was carried out by real-time PCR. The results showed that E-cadherin mRNA in shHOTAIR SKOV3 cells from injected tumor-bearing mice was higher than that in control cells, while the TGF-β1 and zeb1 expression was lower than that in control SKOV3 cells. The difference was statistically significant (* *P* < 0.05), as shown in Fig. 7b, c, d.

## Discussion

LncRNA HOTAIR is highly expressed in epithelial cancer. It indicates poor prognosis and multiple metastases in patients [7, 8], but its related mechanisms have rarely been thoroughly studied. Our in vitro and in vivo research results indicate that low expression of the lncRNA HOTAIR can lead to increased E-cadherin expression in EOC SKOV3 cells, while the expression of zeb1 and TGF-β1 decreases. In addition, low expression of the lncRNA HOTAIR also reduces the invasiveness of EOC SKOV3 cells to matrix gel and their tumorigenicity in nude mice. Zinc finger E-box binding homologous box 1 (ZEB1) is considered a transcription factor and a suppressor of cell adhesion molecules and cell polarity-related genes. During the EMT process, it inhibits genes involved in maintaining epithelial genes (E-cadherin) and activates the transformation to mesenchymal phenotype. The abnormal expression of ZEB1 in many cancers promotes progression, and high ZEB1 expression promotes the progression of gynecological cancer. Therefore, ZEB1 is an important regulatory factor that accelerates the occurrence, development, invasion, and metastasis of tumors, as well as the EMT process [9]. Epithelial-mesenchymal transition is the process of polar epithelial cells transforming into active mesenchymal cells and gaining invasion and migration ability, which exists in multiple physiological and pathological processes in the human body. EMT involves multiple signal transduction pathways and complex molecular mechanisms, which are related to calcium-linking proteins, growth factors, transcription factors, and the microenvironment. EMT is closely related to the invasion and metastasis of tumor cells [10]. Increased zeb1 expression can lead to E-cadherin promoter overbinding zeb1 and inhibit E-cadherin transcription and translation [11, 12]. Transforming growth factor-β (TGF-β) belongs to a newly discovered group of the TGF-β superfamily that can regulate cell growth and differentiation. Various cells in the body can secrete TGF-β. TGF-β may be detected in almost all tumor cells. It can promote the expression of zeb1 in tumor cells, thereby inhibiting the expression of E-cadherin, and promoting N-cadherin and vimentin, contributing to cellular movement, and enhancing cancer epithelial-mesenchymal transition and movement [13, 14]. In our study, down-regulation of the lncRNA HOTAIR in SKOV3 epithelial ovarian cancer cells reduced the expression of zeb1 and TGF-β1. This suggests that lncRNA HOTAIR downregulation might also reduce TGF-β1 secretion from cancer cells. It has been confirmed that TGF-β pathways coordinate many cellular processes, including cell growth, differentiation, migration, invasion, and extracellular matrix remodeling. This pathway and its components exhibit dysregulation in various tumors. In normal cells and early tumors, TGF-β signal transduction inhibits tumor formation by inhibiting cell growth and apoptosis. However, cells can capture TGF-β mutations in its downstream effector factors, resulting in resistance to these anti growth effects. At this point, TGF-β signal transduction becomes a tumor promoting factor. Low levels of TGF-β can inhibit tumor growth, while high levels of TGF-β can accelerate tumor growth. Further research is needed on the specific mechanisms involved.

The high expression of lncRNA HOTAIR in epithelial cancer can promote cellular EMT, metastasis, and CSC formation, and is positively related to the poor prognosis of tumor patients [15–17]. Cancer stem cells are tumor cells with self-renewal ability and can produce heterogeneous tumor cells. Cancer stem cells play an important role in tumor survival, proliferation, metastasis, and recurrence [18]. The lncRNA HOTAIR may be a significant driving regulatory factor for malignant tumor proliferation, EMT, and metastasis [17]. LncRNA HOTAIR high-expression can inhibit the expression of E-cadherin by recruiting histonemethylase EZH2 to mediate H3K27 trimethylation, thereby affecting the progression of nasopharyngeal carcinoma [19]. HOTAIR can promote colon cancer progression by negatively regulating miR-34a [20]. TGF-β, snail, ZEB and twist families are all involved in the process of epithelial-mesenchymal transformation, migration, and metastasis of tumors [21]. Further in-depth research is needed on the interrelationships between these proteins and molecules and lncRNA HOTAIR.

Our study found that compared to the scramble control, the E-cadherin mRNA in shHOTAIR SKOV3 cells increased. And the increased expression of E-cadherin led to increased adhesion and reduced migration and invasion of epithelial cancer cells [22]. Fluorescence quantitative PCR and immunohistochemical methods were applied to check the expression of ZEB1. The ZEB1 expression in downregulated HOTAIR SKOV3 cells is lower than that in the control, and the decreased ZEB1 expression will lead to an increase in E-cadherin expression in cancer cells. Our research elucidates the expression relationship between lncRNA HOTAIR, TGF-β1, ZEB1, and E-cadherin in epithelial ovarian cancer. But we have the limitations of research funding and experimental conditions in the study, as we were unable to conduct a transcriptome-wide exploration to thoroughly investigate the effects of downregulated lncRNA HOTAIR on EOC SKOV3 cells. The research object of transcriptome sequencing is the sum of all RNA that a specific cell can transcribe in a certain functional state, including mRNA and non coding RNA [23]. It is currently a powerful tool for in-depth research on transcriptome complexity. Transcriptome sequencing technology based on high-throughput sequencing platforms can comprehensively obtain transcriptome information of specific tissues or organs of species, enabling research on gene expression levels, discovery of new transcripts, and structural variation of transcripts [24]. We explored the relationship between lncRNA HOTAIR and three important molecules, TGF-β1 and ZEB1 and E-cadherin, which are associated with cancer progression, metastasis and epithelial-mesenchymal transformation (EMT). The expression of these genes can explain some issues and situations. In the future, when we receive more funding and project support, we will continue to explore more mechanisms and compare transcriptome differences of different transfected cells and tumor masses on EOC SKOV3 cells.

## Conclusions

In brief, in this research, lncRNA HOTAIR downregulation caused a decrease in zeb1 and TGF-β1 but an increase in E-cadherin in epithelial ovarian cancer SKOV3 cells. Additionally, the matrix gel invasion and tumorigenic capability of EOC SKOV3 cells were weakened. These foregoing experimental results indicate that lncRNA HOTAIR may become one new and effective target for epithelial ovarian cancer molecular targeted therapy.

### Supplementary Information

Additional file1 (DOCX 45 KB)

## Data Availability

The data used and/or analyzed during the current study are available from the corresponding author upon reasonable request.
