# Supplementary material for: LncRNA HOTAIR down-expression inhibits the invasion and tumorigenicity of epithelial ovarian cancer cells by suppressing TGF-β1 and ZEB1
Source: Discov Oncol. 2023 Dec 9;14:228. doi: 10.1007/s12672-023-00846-5 (PMC10710393; doi:10.1007/s12672-023-00846-5)
Supplement: Supplementary file 1 — Additional file1 (DOCX 45 KB) [file 12672_2023_846_MOESM1_ESM.docx]

Additional file Information

# Additional file 1 Figures

**
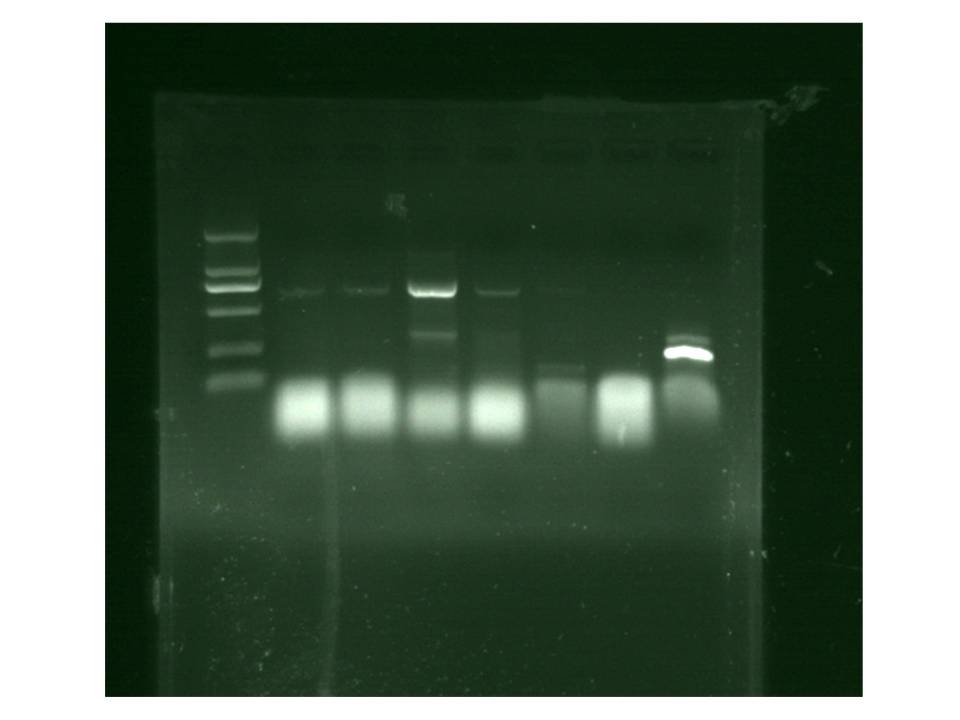
**

Additional file **Figure 1. the full length gel of RT-PCR of lncRNA HOTAIR detection.** The image was the original, unprocessed version.
